# Supplementary material for: Are carbon emissions trading and green financial instruments synergistic? -Comprehensive quantitative research based on content analysis
Source: PLoS One. 2024 Mar 7;19(3):e0298601. doi: 10.1371/journal.pone.0298601 (PMC10919601; doi:10.1371/journal.pone.0298601)
Supplement: S1 Data — (ZIP) [file pone.0298601.s001.zip › Data/地级市Z_F工作报告.zip!/┴╔─■/║∙┬1⁄2╡║╩╨/└┤╫╘╣1⁄2╓┌║┼í╛┬φ┐╦╩2╛▌═°í┐_2013.docx]

各位代表：

现在，我代表市人民政府向大会报告工作，请予审议，并请各位政协委员和其他列席同志提出意见和建议。

一、2012年工作回顾

刚刚过去的2012年，是我市发展史上极不平凡的一年。面对世界经济复苏放缓、国内经济下行的严峻形势，在市委的正确领导下，全市人民解放思想，直面挑战，顽强拼搏，经济实现企稳回升，社会事业繁荣进步，人民群众幸福指数不断攀升，圆满完成了市四届人大三次会议确定的各项目标任务。

预计2012年，全市地区生产总值715亿元，增长10%；公共财政预算收入84亿元，增长19.4%；固定资产投资580亿元，增长30.5%；社会消费品零售总额318亿元，增长16%；城镇居民人均可支配收入23400元，增长16%；农民人均纯收入9170元，增长16%。这充分表明，我市实现了经济发展与居民收入同步增长，沿海经济强市建设取得了重要成果！

一年来，我们主要取得了九个方面的业绩：

第一，沿海开放强力推进。坚持集中精力抓招商，层层签订责任状。健全专业招商队伍，出台重大招商项目联席会议制度、招商引资引荐人奖励制度等政策措施。领导亲自包装项目，十赴江南和欧美港台，招商引资再创新高。全年新批外商投资企业82家，增长36%；实际利用外资5.3亿美元，增长19.3%。亿元以上内资项目落地145个，增长30%；引进内资230亿元，增长35.3%。特别是与德国林德集团签订投资25亿元的工业气体项目，实现了对世界500强企业招商引资的新突破。

园区发展显现活力。完善管理体制，下放管理权限，收入分配制度改革扎实推进。13个园区完成固定资产投资386亿元，增长25.1%。110平方公里起步区实现“七通一平”，200万平方米标准化厂房竣工。新引进项目164个，总投资318亿元，项目总数突破1000个。园区地区生产总值110亿元，增长20.4%；公共财政预算收入16.6亿元，增长38.3%。

“项目建设年”活动成果显著。全市实施亿元以上项目314个，总投资2097亿元，项目数量和投资规模均列全省第5位。36万吨聚苯乙烯、10万吨糠醇、高岭背靠背二期电网、长志泵业等80个项目建成投产，完成项目投资400亿元，增长33.8%，占固定资产投资的67%。投资130亿元的工业铝型材、投资28亿元的中航黎明装备制造基地等重大项目开工。历经艰辛努力，徐大堡核电被列入国家“十二五”开工备选项目，迎来开工建设的曙光。

第二，工业经济稳中有进。开展“深入基层调研服务”活动，市级领导包扶重点企业、包建重点项目，一企一策解难题，90%的工业企业保持满负荷生产。规模以上工业企业达到340家，总产值 1010亿元，增长10%。产业集群提速发展，聚氨酯、泵阀等6个产业集群企业达到812家，增加70家；销售收入1030亿元，增长25%。精细化工产业高点起步，产业集群销售收入突破500亿元，泳装、海工、有色金属3个产业集群销售收入均突破100亿元。泳装产业集群被评为“中国服装产业十大示范集群”，打渔山园区被认定为“中国泵业基地”。

转型发展步伐加快。实施技改项目35个，完成省技术创新重点项目146项，高新技术产业产值115亿元，单位工业增加值能耗下降3.5%。建成省级企业技术中心17个，获得“专精特新”产品称号26项。中航黎明锦化机、龙源采油机电设备等10家企业被评为“国家高新技术企业”，锦西石化、绥电公司跻身省“百强纳税企业”，渤船重工成为全省第一批“新兴高技术领军企业”。

民营经济整体跃升。实施民营经济腾飞计划和中小微企业成长工程，新建中小企业服务平台5个，小额贷款公司开业13家。规模以上民营工业企业达到320家，占全市的94.1%。民营经济总产值突破1500亿元。八家矿业公司上市在辽宁证监局备案。“蝶姿”、“欧意”等6个泳装品牌荣获2012年度中国服装成长型品牌。

第三，县域经济增势强劲。县域生产总值365.6亿元，公共财政预算收入32.9亿元，固定资产投资339亿元，分别增长28.3%、36.3%和36.7%，分别占全市的51.1%、39.2%和58.5%。成功抵御我市有气象记录以来最强的台风和洪涝灾害，粮食产量130万吨，是历史上第二大丰收年。水果、蔬菜、肉蛋奶和水产品分别达到110万吨、243.9万吨、75.2万吨和45.6万吨，分别增长8%、8.2%、9.1%和6%。

农业产业化和新农村建设加快推进。新建、改建设施蔬菜小区25.3万亩，新增节水滴灌20万亩，新建畜禽标准化养殖小区94个，规模化水产品养殖面积150万平方米，新认定无公害水产品21个。国家地理标志商标达到13个，列全省第1位。农业产业龙头企业达到240家，农民专业合作组织达到1356个。完成农田水利工程 427项，治理水土流失33万亩。10亿元以上农产品深加工项目竣工6个，新开工4个。农产品质量合格率高于全省平均水平，我市“无疫区建设”顺利通过国家验收。兴城、绥中、建昌、连山成为“国家蔬菜产业重点县”。建昌县荣获全国农田水利建设先进县和全国农村集体“三资”管理示范县称号。

第四，城区经济发展提速。启动新一轮城区经济“三年倍增计划”。实施葫兴同城化战略。连山东城新区、龙港商贸金融一条街初步形成规模，城区经济发展空间不断拓展。南票区完成行政区划调整，实现近海发展。

服务业集聚区提档升级。连山、龙港、觉华岛和东戴河服务业集聚区被认定为省级集聚区，总数名列全省第3位。玉皇商城、葫芦山庄跻身全省100家服务业领军企业，连山东城华府、龙港文萃路、觉华岛被列入全省100个服务业重点项目。东戴河同湾康年五星级酒店投入使用，飞天购物广场开业运营。10家商贸流通企业获“辽宁商业服务品牌”称号。

现代服务业不断壮大。交通、辽阳2家银行在我市开业运营，全市商业银行达到14家。金融机构存款余额1062亿元，增长13.4%；贷款余额637亿元，增长12.6%。第二期公司债券通过国家发改委初审。电子商务迅速崛起，全市电子商务网站超过1700家。房地产开发投资132.3亿元，增长64%；商品房销售额82亿元，增长60%，在全省领先。全市服务业增加值290亿元，增长12%；城区服务业增加值149.4亿元，增长13%；服务业增加值占全市GDP的40.6%，有力拉动了经济增长。

第五，文化旅游产业加速推进。长城旅游、红色旅游、温泉旅游和乡村旅游示范板块规划编制完成。17万平方米国际和平广场、5公里沿海观光栈道和“一场五馆”竣工，国家沙排训练基地落户我市，世界温泉部落、首山丽汤、红麦坊温泉旅游项目开工。与省文化厅合作共建19个文物保护项目，兴城城墙入选世界文化遗产申报预备名单，兴城古城保护工程全面启动，建昌东大杖子战国墓葬群被评为“2011年全国十大考古新发现”，改写了“春秋战国时期考古不出关”的历史。

文化创意产业起步发展。规划实施12.5平方公里的邴家湾文化创意和温泉旅游产业园，成功举办第二届中国国际沙滩·泳装文化博览会和第二届打渔山泵阀产品博览会，与53家企业签订投资协议281.3亿元，与58家企业签订购销合同20亿元。“海上传奇·北方创意经济”峰会、消夏音乐节、青田石雕主题展和司徒安雕塑展反响热烈，葫芦山庄成为国家级文化产业示范基地，歌舞剧《虹螺女》获省第十二届“五个一工程”入选作品奖，“比基尼阔步巡游”创吉尼斯世界纪录。全年接待游客突破2000万人次，增长23.4%；旅游收入实现212亿元，增长37%。文化旅游已经成为我市的新型支柱产业。

第六，生态宜居滨城建设步伐加快。新一轮《城市总体规划》修编有序推进，16公里龙湾大街全线贯通，一路华灯纵贯葫兴。“我们的城市·我们的家园”城市综合整治活动取得新成效，启动龙湾公园和龙湾海滨复建工程，实施18个城中村、城郊村改造，新建、改建12处景观喷泉，新增城区绿地47公顷，消除老城区28条小街小巷“摸黑路”。我市成为国家“地理空间框架建设推广城市”和省级卫生城市。

城乡基础设施日趋完善。青山水库大坝成功截流，主体工程基本完工，猴山水库开工建设。与营口港务集团签订合作协议，石河港恢复施工，兴城陆岛交通码头扩建工程开工。绕城公路、新老城区快速干道建设启动，建兴高速公路完成动迁任务，并全面开工，葫六线大修、北站路拓宽等10余条主要公路改造竣工通车。

生态环境持续改善。“国家森林城市”创建起步实施，辽西绿色生态长廊工程竣工，植树造林155.8万亩。青山工程取得优异成绩，“小开荒”还林、超坡地还林、工程封育和矿山生态治理全部完成年度任务，连续两年荣获“省造林绿化先进市”称号。凌河生态治理进展顺利，2个湿地生态公园开工，退田还河1990亩。改造新老城区污水处理厂2座，新建乡镇污水处理厂9座，取消分散锅炉房22座，更换城市生物质燃料茶浴炉188套。环境空气质量优良天数达356天，占全年的97.3%。

第七，民生工程成效显著。始终把保障和改善民生放在重要位置，市直29个部门联合开展了惠民政策落实情况大排查活动，着力解决损害群众利益突出问题，年初市政府承诺的10件民生实事全部兑现。城乡低保和农村五保、孤儿孤老救助标准显著提高，百岁老人长寿补贴增加50%。基本养老、医疗、失业、工伤、生育保险参保率全面提升。农村医疗救助实施“一站式”结算。发放各类惠农补贴3.5亿元。完成农村饮水安全工程331处，解决了10.3万农村群众饮水安全问题。完成国电西部供热工程二期和海滨路集中供热厂建设，集中连片低温区基本消除。

群众生活条件日益改善。农村分散供养五保户、低保户和低保边缘户房屋改造工程全面启动，完成城镇保障性安居工程年度建设任务，全省规模最大的国有工矿棚户区改造工程基本完成，解决了14万人的住房困难问题，我市荣获“全国城乡住房建设先进单位”称号。广播、电视综合覆盖率分别达到98.4%和97.6%。新增5条公交线路，公交IC卡开始使用，城区老年人享受免费乘坐公交车福利政策。完成长途运输公司改制，标志着我市公共交通改革取得成功。加强校车安全整治，222辆备案校车投入使用，保障了中小学生上学安全，受到广大学生家长的好评。

第八，各项社会事业全面进步。实施人才强市战略，引进海外研发团队5个。社会保障水平再度提升，城镇实名制就业5.5万人，普惠制培训2.3万人，农村劳动力转移就业32万人，零就业家庭动态管理为零，城镇登记失业率为3.2%。全市新农保参保108.3万人，增长26%；常住人口新农合参合率105.4%，农业人口参合率94.5%。优化和整合教育资源，“普九”工作提前实现“双高”目标，渤海船院新校区开工建设，辽宁财贸学院成为“辽宁社会科学院理论研究基地”。公共文化事业不断进步，读书节、文化大讲堂等10大品牌文化活动活跃滨城。体育事业长足发展，我市运动员进入世界围棋锦标赛四强、荣获全国沙排巡回赛总决赛冠军。人口和计划生育综合改革富有成效，荣获省示范市称号。国防动员、双拥共建成绩显著，荣膺“全国双拥模范城”四连冠。国家安全、外事侨务、民族宗教、行政审批、新闻出版、对口支援、公积金管理和海关、审计、统计、人防、质监、档案、供电、气象、地震、对台、海防、残联、红十字会等工作取得新成绩。

第九，民主法制和精神文明建设再上新台阶。自觉接受人大的法律监督、工作监督和政协的民主监督，坚持定期向市人大和市政协报告、通报工作，认真听取和采纳人大代表、政协委员的意见、建议，主动加强与民主党派、工商联、无党派人士、各人民团体的联系。全年办理人大代表建议75件、政协提案490件，办复率、见面沟通率、满意和基本满意率均为100%，落实和基本落实率65.1%，比上年提高1个百分点。强化“五大系统”建设，41家单位251项行政审批项目实现网上公开。推进依法行政，“六五”普法和法治城市创建工作取得阶段性成果。健全社会预警体系和应急机制，应急保障能力明显提升。加强和创新社会管理，加大社会矛盾纠纷调处力度，积案化解率达到90%，信访总量下降47%。加强“平安葫芦岛”建设，圆满完成十八大安保维稳任务，社会治安防控体系进一步完善。严厉打击食品药品领域违法犯罪行为，保护了人民群众的身体健康。安全生产“打非治违”工作在全国介绍经验，连续6年荣获“全国安全生产月活动优秀单位”，连续3年荣获省“安全生产工作先进市”，连续7年荣获省“综治先进市”称号！

精神文明建设深入开展。绥中、建昌、连山、龙港等7个单位分获全国、省文明县城、文明城区和文明单位（村镇）荣誉称号。“迎全运、爱家乡、建辽宁”主题教育实践活动丰富多彩，“喜迎十八大、学习十八大”主题文化宣传活动全面推进，市民文明素质和城乡文明程度显著提升。

各位代表！过去一年取得的成绩，是全市人民同心协力、团结奋斗的结果，凝聚着广大干部群众的心血和汗水，饱含了方方面面的帮助和支持。在此，我代表市人民政府，向全市广大干部群众，向各位人大代表、政协委员，向各民主党派、工商联、无党派人士，向各人民团体、驻在单位、驻葫部队、武警官兵、公安民警、离退休干部和社会各界人士，向所有关心支持我市发展的同志们、朋友们，致以崇高的敬意和衷心的感谢！

在肯定成绩的同时，我们也清醒地看到，我们的工作仍然面临不少困难和问题：一是宏观经济环境依然严峻，保持经济企稳向好的压力仍然较大。二是赶超发展的基础还不牢固，经济总量较小、地区之间发展不平衡的问题仍未根本解决。三是园区对全市经济的拉动作用不够明显，体制机制与快速发展的要求还不完全适应。四是农业生产力落后，农村面貌还没有根本改变，农民增收渠道不宽。五是软环境建设仍需进一步改善，一些部门服务意识淡薄，工作效率不高，吃拿卡要现象依然存在。对于这些问题，我们一定高度重视，认真加以解决。

二、2013年工作安排

2013年，是全面贯彻落实党的十八大精神、全面建成小康社会的开局之年。站在新的历史起点上，我们必须坚持科学发展、赶超发展的信念不动摇，坚持改善民生、富民强市的根本不动摇，坚持改革创新、锐意进取的志气不动摇，凝神聚力，扎实苦干，推动经济社会持续健康发展。

今年政府工作的总体思路是：认真贯彻党的十八大、中央和省经济工作会议、市委四届六次全会暨经济工作会议精神，坚持科学发展观，努力转变经济发展方式，实施经济结构战略性调整，努力提高经济增长质量和效益，推动工业和服务业双轮驱动、国有经济和民营经济协调发展、县域和城区经济共同繁荣，加速推进城镇化进程，促进经济持续增长、人民生活持续改善、文化产业和社会事业全面发展，加快建设更加富裕、更加美丽、更加幸福的葫芦岛。

主要预期目标是：地区生产总值增长11%，公共财政预算收入增长16%，固定资产投资增长30%，社会消费品零售总额增长15%，实际利用外资增长20%，引进内资增长30%，外贸出口增长10%，城镇居民人均可支配收入增长15%，农民人均纯收入增长15%。

为实现上述目标，要重点抓好以下九个方面工作：

1、着力推动工业结构调整，促进工业经济提质升级

积极推进工业五项工程。实施企业提升工程，强化对骨干企业的帮扶措施，促进效益提升；推动绥电公司80万千瓦机组节能改造和锦西石化汽柴油质量升级，培育国家级高新技术企业15家以上，高新技术产业增加值占比提升5个百分点，全市规模以上工业增加值增长10%以上。实施项目工程，继续开展“项目建设年”活动，实施投资5000万元以上工业项目359个，总投资1603亿元；重点推进徐大堡核电、千万吨炼油、方大化工甲醇系列产品一体化和渤船重工海洋工程等179个亿元以上工业项目。实施产业集群工程，全市产业集群企业数量力争突破1000家，每个产业集群销售收入增长30%。实施节能和淘汰落后产能工程，淘汰3家高耗能、高污染企业，单位工业增加值能耗降低率达到5%。实施海外并购工程，引导我市企业积极参与海外并购。

规划建设精细化工产业园。制定精细化工产业发展规划，加速推进拜耳集团一体化化工项目落户，促进与亨斯迈、巴斯夫等国际化工巨头的合作。开工建设林德工业气体、10万吨针状焦、聚氨酯软泡等项目，集聚工业经济增长新动力。

加快中小微企业发展。建立创业种子基金，鼓励全民创业。扩大专项支持资金规模，出台普惠减负措施，建立普惠创业辅导机制。加快服务平台建设，加大融资担保力度，推进商业银行扩大向中小微企业贷款的规模，小额贷款公司贷款额突破10亿元。加强对科技成长型中小微企业的扶持，促进中小微企业数量、规模、效益同步提升。

2、着力推动城乡统筹发展，壮大县区经济实力

积极推动县域工业化、城镇化、农业现代化同步发展。引进大田作物新品种100个，新建、改建设施农业20万亩，新建节水滴灌20万亩、现代海洋牧场2万亩，建设万亩粮油高产区20个、畜禽标准化小区70个。加快推进兴城花生、绥中白梨和建昌核桃等“一县一业”发展。开工建设绿宝露等5个10亿元以上农产品深加工项目，雨润食品等4个项目投产。加快县城和小城镇建设，重点推进兴城邴家湾和滨海新区、绥中东戴河新区和六股河新城、建昌凌河新区建设，打造10个明星城镇，城镇化率提高5个百分点。加强新农村建设，新增20个市级新农村示范村。全面实现县域经济“三年倍增”目标。

加快以服务业为重点的城区经济发展。规划以“一主四辅”为核心的服务业集聚区发展格局，推进“金岸龙湾”旗帜型服务业集聚区和连山、东戴河、建昌、南票新城等4个重点服务业集聚区建设，辐射全市14个服务业集聚区协调发展，服务业增加值增长12%。投资105亿元的广东商贸物流城、投资60亿元的滨海金融中心、投资60亿元的隆东商业综合体等重大服务业项目开工，投资10亿元的特易购大卖场、投资5亿元的宏运购物中心等大型商场竣工。加快发展电子商务等现代服务业，培育新的消费增长点。积极引进域外银行业金融机构，民生银行、广发银行葫芦岛分行开业运营，全年各项贷款余额和表外业务融资均增长15%以上。推进房地产业持续健康发展，全市房地产业开发投资、商品房销售额分别增长20%。提升龙湾CBD现代服务业功能，加快南票新城区建设，打造葫兴一体化大城区格局。

3、着力推动文化创意产业成长，建设文化旅游强市

推进文化创意产业发展。编制文化创意产业总体规划，加速文化创意产业园建设。建设“北方风俗文化园”、“北方国际创意会展中心”和“中国比基尼博览中心”。推出“传奇比基尼”文化创意产品，推动满族刺绣等民间工艺产业化，促进影视、乐园、会展等文化创意产业发展。开工建设投资5亿欧元的卡那多斯游艇俱乐部、投资1亿美元的亿湾陆森游艇俱乐部和投资50亿元的莲花山文化产业园项目。办好泳博会和泵博会，塑造特色节会文化。

加快文化旅游资源开发。推进兴城古城、姜女石遗址、建昌古墓葬群和莲花山圣水寺等4大文物本体保护，规划战国古墓遗址公园，启动古城名人故居、圣水寺和锥子山长城维修项目。打响海滨、温泉、乡村、古城、长城和岛屿6个文化旅游品牌，建设觉华岛“中国北方生态旅游岛”，打造龙湾至兴城海滨、九门口至锥子山长城、莲花山至虹螺山、古城首山至邴家湾、绥中海滨等5条精品旅游带。开工建设比基尼广场、小虹螺山景区，完成塔山阻击战纪念馆布展，龙湾至兴城海滨10公里、东戴河6公里观光栈道全线贯通，推进红麦坊温泉旅游度假村、古城丽汤温泉养生谷、香港俊安海洋世界和梦幻海洋城等温泉旅游项目建设，打造具有国际水准、北国风情的滨海旅游度假胜地和旅游目的地城市！

4、着力激发园区创新活力，加快沿海开发开放步伐

大力提升园区承载能力。深化园区用人、分配和管理制度改革，完善灵活的用人机制，实行绩效工资制度，建立自主发展的运行机制。坚持集约节约用地，提高土地利用效率。优化园区基础设施，新增30平方公里“七通一平”，再建200万平方米标准化厂房。

全力推进招商引资。以世界500强、国内500强和行业100强为重点，实行组团式招商、园区化招商，实施重大招商项目攻坚。积极参与国内外展会、全国性论坛，拓宽招商渠道。落实项目包装、落地和缴资责任制，签约项目落地率达到30%。

加快6个省级重点园区项目建设。东戴河新区引进数字技术项目50个以上，固定资产投资148亿元。兴城临海产业区引进新材料项目20个以上，固定资产投资60亿元。高新区引进精细化工项目20个以上，固定资产投资20亿元。打渔山园区引进泵阀项目30个以上，固定资产投资70亿元。北港工业区引进项目20个以上，固定资产投资20亿元。觉华岛旅游度假区重点推进11个项目，固定资产投资10亿元。同时，海洋工程园区等其他7个园区引进项目和固定资产投资都要实现重大进展。

5、着力推动生态文明建设，打造美丽葫芦岛

完善城市设施。加快推进葫芦岛港和石河港建设，规划建设临港产业区，实现水陆交通与园区的更好对接。实施老区兴工路、天化街改造。启动全市“四横五纵”公路网工程，开工建设新老区快速干道，加快建兴高速公路建设，改扩建5条重点公路。开工建设投资31亿元的大唐热电项目，推进城区拆小并大、供热计量和节能改造工作，逐步实现集中、高效供暖。

提升城市品位。深化“我们的城市·我们的家园”活动，城市建设向老城区倾斜，向农村延伸。开展“绿满滨城”行动，建设龙湾生态公园和海滨公园，完善城市道路绿化带。开展“温馨滨城”行动，加快18个城中村、城郊村改造进程，完成年度动迁和建设任务；新建饮水安全工程90处，彻底解决我市饮水安全问题。开展“霓虹滨城”行动，城市亮化向高雅提升、向小街小巷延展。

优化生态环境。全力创建国家森林城市，建立全域生态绿化体系。加快青山工程建设，加强矿产资源保护和农村生态环境治理，造林育林47.6万亩，矿山生态治理5633亩。加快碧水工程建设，实施茨山河上游综合治理，改扩建新老区污水处理厂，日处理能力17万吨；编制大小凌河流域生态带、旅游带规划，治理水土流失24万亩；严格实施海洋功能区划，加大资源管控力度，大力发展海洋经济。加快蓝天工程建设，推进“一县一热源”，加强大气污染防治，确保空气质量稳定达标。

6、着力推动民生改善，让发展成果更多惠及全市人民

加快改善民生步伐，力争到2016年，实现地区生产总值和城乡居民收入比2010年翻一番，提前实现全面建成小康社会目标。今年，要全力办好10件惠民实事：

一是帮助农民致富，鼓励农民建设蔬菜标准园，全市建设10个，每个补助100万元；培训农民技术骨干3000人，每人补助500元。二是创建城区标准化普惠性幼儿园20所，改建和增设农村普惠性幼儿园30所。三是加强教育强县（区）建设，推进义务教育均衡发展，创建农村义务教育标准化学校74所。四是建购并举，改造农村危房5200户，让农村五保户、低保户和低保边缘户逐步住上安全舒适的房子。五是推进城乡公交一体化，新增公交车50台，开通新线路3条，新设公交专用道10公里。六是实施“城市医生支援农村”工程，组织10家城市医院、80名城市医生，对口支援10家农村卫生院，提高农村医疗水平。七是提升高龄老人补贴标准，90岁以上老人补贴由50元/月提高到100元/月，百岁以上老人长寿补贴由300元/月提高到600元/月。八是实施文化惠民工程，综合文化站实现乡镇全覆盖，公共图书馆、文化馆、博物馆免费开放。九是加强扶贫开发，扶贫标准提高到2500元/人，确保24.4万人稳定脱贫。十是加强青山水库上游生态保护，开工建设水安全和输水配套工程，确保具备供水条件，解决城区供水不足问题。

7、着力推动社会事业全面发展，提升公共服务质量

深化行政体制改革，分类推进事业单位改革。努力扩大就业，新增实名制就业3.6万人，城镇登记失业率控制在3.8%以内。提高新农合人均筹资标准和城乡低保标准，基本养老、医疗、失业保险实现应保尽保。加强科技创新，促进科技成果的转化和应用。实施渤海船院主体搬迁，整合化工技校和工业学校等中职学校资源，打造高、中职教育示范区。推进县级医院改革，加快市中心医院二部改扩建，政府办基层医疗机构基本药物制度实现全覆盖。实行免费孕前优生健康检查，人口出生率控制在6.67‰以内，出生人口性别比控制在103~107之间。完善食品安全溯源机制，健全食品药品监管制度。开展文化下乡、文化广场等活动，丰富全民文化生活。全力办好足球、蹦床等第十二届全运会有关赛事，组织好火炬传递活动，提升葫芦岛举办大型体育赛事的影响力。同时，做好双拥共建、质量技术监督、防灾减灾、民族宗教、外事、对台、人防、海防和红十字会、残联等工作。

8、着力加强精神文明和民主法制建设，维护社会和谐稳定

加强民主法制建设。自觉接受人大的法律监督、工作监督和政协的民主监督，诚恳听取各民主党派、工商联、无党派人士和人民团体意见，提高人大代表议案、建议、批评、意见和政协提案办理质量。加强市长热线、市长信箱和政府网站建设，发挥新闻媒体作用，畅通人民群众表达意愿的渠道。进一步开展“六五”普法和法治城市创建工作，推进政务公开和行政监察，自觉接受舆论监督，增强政府工作的透明度和公信力。

加强精神文明建设和社会管理创新。深入贯彻落实党的十八大精神，弘扬社会主义核心价值观，启动“全国文明城市”创建工作。加强诚信体系建设，打造“诚信葫芦岛”。加强信访矛盾排查，完善大调解机制，深入基层解决问题。抓好社会管理创新综合试点，发挥民间组织作用，促进服务和管理向社区倾斜。深化“平安葫芦岛”建设，健全社会治安综合防控体系，争创社会管理综治工作先进市全省“八连冠”和全国“两连冠”。强化安全管理和隐患排查整治，坚决遏制重特大安全事故发生。进一步健全突发公共事件应急管理机制，提高预防预警和处置能力。

9、着力推动行政效能提升，强化政府自身建设

人民对美好生活的向往，就是我们的奋斗目标。我们要以加快发展为己任，尽心尽力做好政府各项工作。

建设创新政府。改进决策程序，推动公众参与决策、监督决策机制建设。强化工作协调，推行部门主办制、联席会议制，提高行政资源效益。优化行政审批流程，规范办事程序。推动管理重心下移，增强基层公共服务能力。

建设高效政府。切实转变工作作风，开短会，讲短话。深入实际，深入群众，解难题，办实事。严格目标责任制，强化绩效考核，激发干部干事激情。落实行政首长负责制，实行权力部门科长定期轮换制，完善政令通畅的执行机制。推进重点工作项目化责任制，实行倒计时工作法，加快工作节奏，限时办结，跟踪问效。

建设清廉政府。全面落实党风廉政建设责任制，加强对重要部门、重点领域的工作监督和审计监督。完善土地招拍挂、工程招投标和政府采购等制度，堵塞工作漏洞。厉行节约，勤俭办事，降低行政成本，把有限的财力用到经济建设、社会发展和改善民生的重点工作上。

建设服务政府。开展“软环境治理年”活动，推进行政问责制，严肃查处吃拿卡要行为，坚决整治不作为现象。对基层和客商的诉求，积极回应，迅速落实。带着感情工作，真诚帮助外来投资者和本地创业者解决实际问题，树立政府和公务员的崭新形象。

各位代表，时代赋予我们庄严使命，人民寄予我们热切期盼。让我们在市委的坚强领导下，在市人大、市政协的监督支持下，团结和依靠全市人民，振奋精神，奋力拼搏，谱写沿海经济强市建设的新篇章！
